# Adalimumab Persistence and Its Biosimilar in Inflammatory Bowel Disease: A Real-World Study

**DOI:** 10.3390/jcm13020556

**Published:** 2024-01-18

**Authors:** María Carmen Fernández-Cano, Antonio Jesús Fernández-Cano, María Mar Martín-Rodríguez, Antonio Damián Sánchez-Capilla, María José Cabello-Tapia, Eduardo Redondo-Cerezo

**Affiliations:** 1 Gastroenterology and Hepatology Unit, Virgen de las Nieves University Hospital, 18014 Granada, Spain; 2 Doctoral Programme in Clinical Medicine and Public Health, Faculty of Medicine, University of Granada, 18012 Granada, Spain; 3 Citizen Innovation Department, Consortium for Developing the Information and Knowledge Society in Andalusia “Fernando de los Ríos”, 18016 Granada, Spain

**Keywords:** adalimumab, drug switching, multiswitching, interchangeability, inflammatory bowel disease, persistence, biosimilar

## Abstract

Adalimumab biosimilar experience is still recent. Interchangeability differences could reduce persistence times. Our goal was to compare biosimilar persistence differences with a reference. A retrospective observational study was performed in three groups divided according to the adalimumab received. The primary outcome measure was persistence, represented with Kaplan–Meier analysis, and we secondarily evaluated security, efficacy, and biomarkers. We obtained approval from the regional ethical committee, and the study was conducted following the Helsinki Declaration as revised in 2013. Data from 104 patients were collected: 50 received the biosimilar, 29 received the reference, and 25 switched from the original to the biosimilar. After a follow-up of 12 months, the biosimilar’s persistence was higher, without differences in mild adverse events per group. In contrast, there were differences in severe events, with the switched group’s frequency being higher. Biomarkers were reduced at similar proportions in all groups, and 43% had a clinical response at week 20 without differences. Adalimumab biosimilars are a valuable option for IBD based on clinical equivalence that are less expensive than the original drug. Their use does not have a detrimental influence on disease, although there are a few nuances in terms of interchangeability. These results support increasing confidence in using biosimilars, thus promoting the better sustainability of health systems.

## 1. Introduction

Biological anti-TNF agents have been considered as a viable therapeutic option for individuals with moderate-to-severe inflammatory bowel disease (IBD). Among these agents, adalimumab has established itself as a prominent treatment choice. Following its patent expiration in the United States in 2016 and in Europe in October 2018 [1], biosimilars were approved by the Food and Drug Administration (FDA) and the European Medicines Agency (EMA) and introduced to the European market in the same year.

However, it is important to acknowledge that biosimilar drugs are not identical replicas of their reference drug [2]. While their authorization is subject to stringent regulatory oversight, there were initial reservations among healthcare professionals regarding the extensive usage of these drugs [3]. Some concerns revolve around the potential molecular differences between the reference drug and its biosimilar counterpart. One particularly contentious issue is the concept of extrapolation, which entails approving a biosimilar for all indications for the reference product without conducting specific clinical trials for each indication individually [4]. The biosimilars employed in this study (ABP 501, GP2017, FKB327, and SB5) had not been previously tested for IBD, as they were marketed after being tested in rheumatological pathologies according to the aforementioned concept of extrapolation [5,6,7,8]. It is crucial to note that although all immune-mediated inflammatory diseases (IMIDs) share a common pathophysiological basis [9,10], not all drugs have an equivalent therapeutic effect on different diseases.

Relatively few real-life studies have investigated the switch to adalimumab biosimilars [11,12,13,14,15,16,17,18,19,20], and a majority of these studies have not included a comparison group of patients receiving the original treatment. The present study was aimed to address this research gap by comparing the time of persistence between adalimumab biosimilars and the reference drug in a cohort of patients, including a mixed group that made the switch from the originator to the biosimilar. Although earlier studies by Derikx L. et al. [16] and Ribaldone D et al. [18] utilized different biosimilars (SB5 and ABP 501), in our center, a total of four biosimilars were used during the follow-up period (ABP 501, GP2017, FKB327, and SB5), with instances where the same patient received at least three different biosimilars. Moreover, the objective of this study was to assess safety, clinical response, and biomarker changes across various patient cohorts. Evaluating safety and tolerance is of utmost importance, particularly when multiple treatment switches are involved.

According to the European Medicines Agency (EMA), interchangeability refers to the ability to safely switch between therapeutically equivalent drugs in clinical practice [21]. This determination is based on the absence of significant differences identified through a dedicated pharmacovigilance program [22], and the decision regarding interchangeability rests with individual European national governments [23]. In contrast, the U.S. Food and Drug Administration (FDA) imposes additional regulatory requirements for interchangeability, including the need for specific clinical switching studies. At present, only one monoclonal antibody has achieved interchangeable status as recognized by the FDA (BI 695501, Boehringer Ingelheim on 15 October 2021) [24,25].

In conclusion, this study is aimed to provide insights into the persistence of adalimumab biosimilars compared with the reference drug in patients with IBD over an extended follow-up period and to add particular insight into what happens when multiple treatment changes are involved. Persistence serves as a valuable parameter for evaluating therapeutic benefits, defined as the duration between the initiation and discontinuation of an agent [26]. Furthermore, this study is aimed to contribute to the existing knowledge and understanding of safety, clinical response, and biomarker changes across different patient cohorts.

## 2. Materials and Methods

This retrospective observational study was conducted at Virgen de las Nieves University Hospital in Granada, Spain, between June 2018 and June 2020. The study included patients enrolled in the adalimumab local registry who received treatment with adalimumab. The data collected for analysis were obtained from the digital clinical records and included clinical, demographic, and serum parameters.

All recruited patients had moderate-to-severe inflammatory bowel disease (IBD) and were treated with adalimumab. The total follow-up period extended for 48 weeks from the initiation of therapy. Patients who did not comply with the prescribed visit schedule or were lost to follow-up were excluded from the study.

The patients were divided into three groups: Group A consisted of patients treated exclusively with the biosimilar form of adalimumab, Group B comprised patients who only received the original adalimumab, and Cohort C (referred to as the “mixed group”) included patients who initially received the reference adalimumab and switched to the biosimilar form. The decision to switch patients from the reference adalimumab to the biosimilar was made by a committee of experts at our center, which included physicians from our unit, regardless of the patients’ clinical status.

The following variables were included in the analysis: demographics (age, sex); toxic habits (smoking, grams of alcohol consumed per day); clinical characteristics (duration of IBD, type of IBD—ulcerative colitis [UC] or Crohn’s disease [CD]); Montreal classification; number of previous surgeries; presence of extraintestinal manifestations; dependence on corticosteroids; previous use of biological treatments; concomitant medications with adalimumab; type of adalimumab administered (biosimilar, original, or both); multiple biosimilar switches; duration of treatment (up to 48 weeks); disease activity measured by the Harvey–Bradshaw Index (HBI) in CD [27] or the partial Mayo score (pMayo) in UC [28] at weeks 0, 10, and 20; clinical response at weeks 10 and 20; adverse events and their severity; need for treatment discontinuation; number of hospital admissions and surgical interventions during the follow-up period; and biochemical parameters including serum C-reactive protein (CRP), hemoglobin (Hb), and fecal calprotectin (FC) at weeks 0, 10, and 20; and baseline albumin levels.

Statistical analysis was performed using R Studio version 4.0.3. Descriptive analysis was conducted, with continuous variables presented as means or medians (depending on the presence of extreme values) with a 95% confidence interval and categorical variables presented as frequencies and percentages. The chi-square test was used to determine significant differences between categorical variables, and Student’s t-test was used for normally distributed quantitative variables. A *p*-value of less than 0.05 was considered statistically significant. Persistence of treatment was assessed using Kaplan–Meier analysis, and the differences in persistence among the groups were compared using the log-rank test.

### Ethical Statements

The present study received ethical clearance from the Ethical Committee for Biomedical Research of Andalusia, with the study code designated as TFM-EAVB-2020 and the committee internal code noted as 2349-N-20. Our research adhered to the Helsinki Declaration, last revised in 2013, in all aspects of its design and execution [29].

## 3. Results

### 3.1. Demographic Characteristics and Groups of Patients

**Figure 1:**
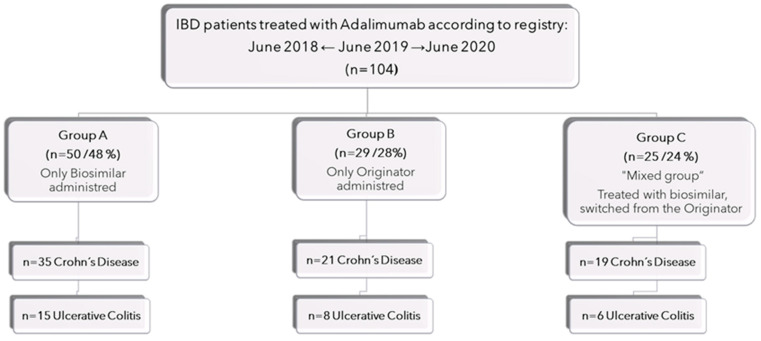
Flowchart of the patient cohorts included in the study.

A total of 104 patients were included in the study and received three different treatment regimens. Among them, 50 patients, referred to as Group A, exclusively received the adalimumab biosimilar, accounting for 48% of the total sample. Another 29 patients, categorized as Group B, only received the reference drug, constituting 28% of the sample. Lastly, 25 patients initially started the treatment with the biosimilar but later switched to the originator drug, making up 24% of the sample and forming Group C. The distribution of patients among these groups and the percentages of patients with Crohn’s disease (CD) and ulcerative colitis (UC) are depicted in Figure 1. Out of the entire sample population, it was observed that seven patients (6.73%) were administered more than two different biosimilars. Within this group, four patients belonged to Group A, accounting for 8% of this specific group, and three patients were from Group C, constituting 12% of the same group. Furthermore, Group C included a subgroup consisting of four patients who initially received treatment with the original adalimumab, transitioned to the biosimilar, and subsequently reverted back to the initial therapy due to either the deterioration of the underlying condition or adverse reactions. This subgroup constituted 16% of the overall mixed group population. Additionally, three patients from Group C were compelled to switch from the biosimilar adalimumab to the reference treatment due to adverse reactions, representing 12% of the mixed group.

The average age at the commencement of treatment was 41.9 years, with a 95% confidence interval (CI) ranging from 39.13 to 44.79. Additionally, the male-to-female ratio was 1:1.

### 3.2. Clinical Features

The average duration of the disease before commencing adalimumab treatment was 94.73 months, with a 95% confidence interval ranging from 76.62 to 112.83 months. There were no significant differences observed in this duration across the three groups (*p*-value = 0.09). Approximately 33.7% of patients had previously undergone surgery, and the distribution of this characteristic was similar among the three treatment groups. Additionally, there were no differences observed in other baseline characteristics, including the proportion of patients who had received prior biological therapies (anti-TNF-alpha non-naive). Disease activity indices were assessed based on the HBI/Mayo score, and no differences were observed between the groups at baseline or in the biochemical activity parameters. These factors contributed to the homogeneity and comparability of the groups. In our total population, Crohn’s disease accounted for 73.1% of cases and ulcerative colitis comprised 26.9%. The distribution of disease type was homogeneous across the three treatment groups. Approximately 23.7% of patients had perianal disease, and 25% had associated extraintestinal manifestations (EIMs), with rheumatological disease being the most prevalent (61.5%). Table 1 provides a comprehensive overview of the demographic and clinical features of the study cohorts.

### 3.3. Aspects Related to Treatment

In order to assess clinical response, specific criteria were applied. For patients with Crohn’s disease (CD), a reduction of three or more points in the baseline Harvey–Bradshaw Index (HBI) score was considered indicative of response at week 0. For patients with ulcerative colitis (UC), a reduction of two or more points in the baseline partial Mayo (pMayo) score was considered indicative of response at week 0.

At week 10, a total of 46 patients (44.23%) displayed clinical response, while at week 20, a total of 35 patients (33.65%) exhibited clinical response (as shown in Table 2).

At week 20, the global loss of response to adalimumab was found to be 10.58%. Among the different groups, the mixed group (C) exhibited the highest rate of loss of response, with a decrease of 50.5% at this time point.

Regarding concomitant treatments, 50% of the sample received additional treatment in conjunction with the biological treatment at some point during the study. Group B had the highest proportion of patients receiving concomitant drugs, with 16 out of 29 patients (representing 65.51% of the group). Groups A and C had lower proportions of patients treated with concomitant drugs, with 50% and 44%, respectively. Specifically, 6.73% (*n* = 7) of the total sample were on combined treatment with an immunomodulator (azathioprine): two patients belonged to Group A (28.7%), four patients belonged to Group B (57.1%), and one patient belonged to Group C (14.2%).

In terms of monitoring drug levels and immunogenicity, 51.9% (*n* = 54) of patients had their serum adalimumab levels and antibodies measured at some point during follow-up. Adequate or above-upper-limit serum adalimumab levels were observed in 72.2% (*n* = 39) of patients, while 27.8% (*n* = 15) of patients had infra-therapeutic levels. Anti-adalimumab antibodies were absent in all patients except for 3 out of the 54 patients (5.5%). Two of these patients belonged to the reference adalimumab group and one belonged to the mixed group. In all cases, antibody levels were measured during the maintenance phase of treatment. Analyses of baseline CRP and FC values revealed similar inflammatory loads among the different groups (Table 2).

### 3.4. Treatment-Emergent Adverse Event (TEAE)

Treatment-emergent adverse events (TEAEs), encompassing both mild and severe treatment-related adverse reactions, were observed during the 48-week follow-up period (see Table 3).

In Group A, a ratio of 28% was observed for treatment-emergent adverse events (TEAEs), with the majority falling into the category of mild TEAEs (22%). Conversely, Group B exhibited a total proportion of 27.58% for TEAEs, of which a higher percentage consisted of severe adverse events (17.24%) as opposed to mild ones (10.34%). Notably, Group C demonstrated a higher overall percentage of patients experiencing a TEAE (60%), with more than half of this proportion suffering from a severe adverse effect (36%).

#### 3.4.1. Mild Adverse Reactions

**Figure 2:**
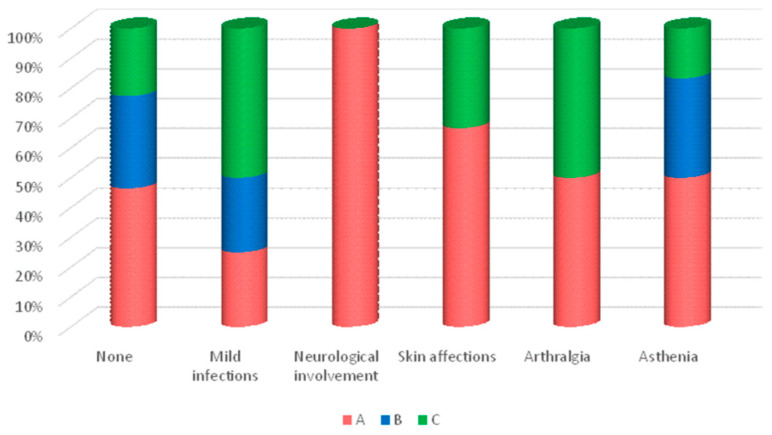
Percentage of contribution to total mild TEAEs by group.

Adverse reactions related to administering the drug that did not require its withdrawal were considered mild because they did not cause a relevant clinical impact on the patient and they did not require specialized care or to visit the emergency room. It is important to note that throughout the one-year follow-up period, 84 patients did not exhibit any adverse reactions. However, a total of 20 patients (19.23%) experienced mild adverse reactions (Figure 2). Among these cases (representing 20% of the mild adverse events), four instances manifested as mild infections, including a urinary tract infection, community-acquired pneumonia without criteria for hospitalization, Campylobacter jejuni infection, and recurrent otitis media.

Notably, three patients, accounting for 15% of mild treatment-emergent adverse events, exhibited neurological involvement. The observed neurological symptoms consisted of headaches, and all three patients received treatment with the identical adalimumab biosimilar (SB5). An additional three patients (15% of mild treatment-emergent adverse events) demonstrated skin involvement, specifically two cases describing skin reactions at the puncture site and one case displaying eczematous lesions in sun-exposed areas. These three patients were administered treatment with the biosimilar, SB5.

Concerning other manifestations, mild joint involvement was observed in four cases, all of which presented with arthralgias lacking a specific pattern (representing 20% of mild adverse events). Six patients, accounting for 30% of mild treatment-emergent adverse events, reported experiencing asthenia on the days of adalimumab administration. Among these six patients, four were receiving treatment with the biosimilar while the remaining two were receiving the original product. Overall, treatment Group A (exclusively treated with the biosimilar) exhibited the highest number of mild adverse reactions, totaling 11 cases. However, these differences failed to achieve statistical significance (*p* = 0.054).

#### 3.4.2. Severe Adverse Reactions

**Figure 3:**
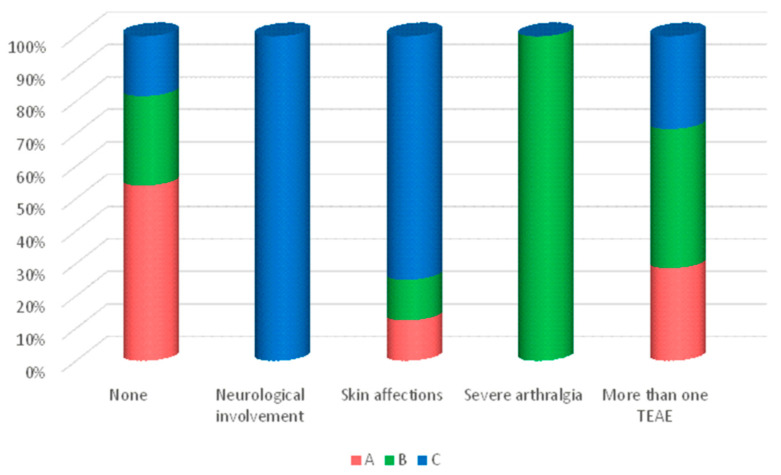
Percentage of contribution to total severe TEAEs by group.

Severe adverse reactions were defined as deleterious effects directly linked to the drug, resulting in therapy discontinuation, hospital admission, or emergency/specialized care intervention. Out of a total of 104 patients, 87 experienced no severe adverse reactions while 17 individuals suffered from such events (Figure 3).

One patient presented with severe neurological symptoms, including paresthesia in the lower limbs and significant muscle weakness, resulting in the discontinuation of treatment. This patient was part of the mixed group and had been on the biosimilar treatment for 20 weeks, receiving multiple different biosimilar brands during that period.

In terms of other severe reactions, eight patients (47%) experienced skin conditions, such as pain at the injection site (n = 4, 3 of whom were in Group C), and two patients developed maculopapular rashes following the administration of the biosimilar, which did not reoccur after switching to the original drug. Additionally, one patient developed hidradenitis suppurativa, requiring a change in treatment to Ustekinumab. The remaining patients in this group had injection reactions to the original adalimumab and had previously experienced infusion reactions with another anti-tumor necrosis factor (TNF) medication, infliximab. The mixed group (C) had the highest proportion of severe reactions, with a total of nine treatment-emergent adverse events (TEAEs). A statistically significant difference in the occurrence of severe TEAEs was observed among the three groups (*p* = 0.009).

### 3.5. Study of Drug Survival (Persistence)

**Figure 4:**
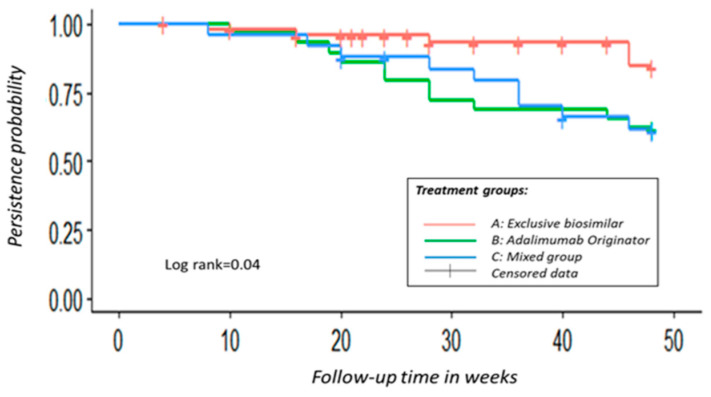
A Kaplan–Meier curve is utilized to demonstrate the persistence function of adalimumab among various treatment groups.

Drug persistence was evaluated by calculating the discontinuation rate and the duration of treatment until discontinuation during the 48-week follow-up period. The overall survival rate of adalimumab treatment after 48 weeks was 80.2% in Group A, 62.1% in Group B, and 61.3% in Group C, as depicted in Figure 4 using the Kaplan–Meier curve.

Throughout the entire duration of the follow-up, a total of four patients (8%) from Group A, eleven patients (38%) from Group B, and nine patients (36%) from Group C discontinued treatment. The reasons for discontinuation among all groups were primarily due to either a loss of response or the development of severe adverse effects. This group also included those individuals in Group C who abandoned the biosimilar treatment to switch to the original adalimumab.

Similar trends in persistence were observed among the three groups during the initial weeks until reaching week 16. Beyond this point, both Groups B and C exhibited more pronounced decreases in persistence, with Group B displaying a slightly more notable decline. Eventually, around week 42, these two groups began to converge, and by week 48, Group C exhibited lower persistence.

Furthermore, Group A, exclusively comprising patients on the biosimilar, demonstrated higher and more consistent persistence compared with the other groups. Notably, significant differences were observed in the survival functions among these groups (*p*-value = 0.04). It is important to mention that the amount of censored data varied across the groups, with Group A having 14 censored data points, Group B having none, and Group C having four. These censored data points refer to patients who did not experience the event of discontinuation during the follow-up period. Specifically, these patients were recruited later and therefore did not have sufficient time to reach the 48th week. This occurrence is typical in dynamic cohorts within survival studies (see Table 4 for the further analysis of these data).

## 4. Discussion

Drug persistence is a crucial parameter that is influenced by multiple variables and provides important information about the clinical outcome of therapy. Therefore, a comprehensive understanding of the factors contributing to drug persistence is vital for optimizing treatment continuity. However, the evaluation of drug persistence has not been a primary focus in most biosimilar studies conducted thus far. The majority of current research focuses on assessing the clinical efficacy and safety of switching from an originator drug to a biosimilar [12]. Limited research exists specifically on adalimumab biosimilars used exclusively for inflammatory bowel disease, as most studies have been conducted for other pathologies such as rheumatoid arthritis [30] or psoriasis [31]. Although some studied have included small groups of patients with inflammatory bowel disease [32], further evidence is needed in this specific context.

It is important to highlight a relevant clinical trial in this regard, namely VOLTAIRE-CD [33]. This multicenter, randomized, double-blind study was conducted in various countries in Europe and the USA, focusing on patients with Crohn’s disease. The trial compared the efficacy and safety of the original drug to a single biosimilar (BI 695501) without considering broader parameters such as drug persistence or survival. Additionally, there are limited data available on the use of multiple switches with adalimumab biosimilars. Some studies have compared different biosimilars with each other, but they only involved a single brand of biosimilar per patient [19]. The closest was a study by Derikx L. et al. [16] in which the efficacy, safety and persistence between original ADA and SB5 (biosimilar) were studied; the study included a small percentage of patients (3.1%) who made a double switch from SB5 to other biosimilars.

In comparison to the existing literature, our study has unique strengths. We conducted a comparative analysis with a group of patients exclusively treated with the original adalimumab, and we included a higher proportion of patients who underwent multiple switches to different biosimilars. Our multiswitched group constituted 6.73% of the sample, twice the proportion of the study by Derikx L.et al. [16]. This has provided us with more comprehensive information; however, we acknowledge that further research is still needed in this area.

In our cohort, we found that drug persistence was higher in the group that initiated biosimilar therapy alone and continued with it, while the mixed group (C) and the original adalimumab group (B) had similar persistence results. When analyzing drug persistence, it is essential to consider various factors that can influence it, such as the nocebo effect; differences in the subcutaneous administration devices used (with or without citrate, as analyzed by Bergman et al. [32]); variations in nursing education; heterogeneity among groups in terms of gender, age, and baseline disease characteristics; and the concurrent use of adalimumab with immunomodulators. Moreover, the issue of interchangeability between the original drug and the biosimilar(s), whether it be a single or multiple switching, also plays a crucial role. We discuss the presence or absence of these specific elements in our cohort in the following sections.

### 4.1. The Nocebo Effect

The nocebo effect refers to the negative effect caused by a drug that is induced by the patient’s expectations. It is not related to the physiological action of the drug but rather arises from the psychosocial context or the therapeutic environment conditioned by the mind and body.

Based on the findings of our study, we contend that the observed results were not influenced by the nocebo effect. Initially, one might deduce that the disparities in persistence could be partially ascribed to varying degrees of the nocebo effect among the groups, particularly evident in the mixed group. However, based on the separate examination of the three groups, it is our belief that the nocebo effect did not play a significant role in Group B, as those patients exclusively used the originator drug, which typically does not instill doubt in patients. Similarly, we posit that Group A, which exclusively used the biosimilar, was unaware of the existence of the branded drug and had not previously utilized it, was not considerably impacted by the nocebo effect either. Although we proceed with caution, it is plausible to consider a partial influence on the results if patients were cognizant of receiving an equivalent drug rather than the reference drug. However, given their lack of prior exposure to the original drug, the potential for such influence was minimized.

Consequently, let us focus on the mixed group (Group C). These individuals had previously experienced positive outcomes with the original adalimumab and were subsequently switched to a biosimilar, which could have affected the results. Nevertheless, a thorough examination of our findings is necessary to alter our perspective. If this were indeed the determining factor, we would also anticipate significant distinctions in persistence between Group C and Group B, such that Group C would exhibit lower persistence. However, as illustrated in Figure 4 and Table 4, this was not the case. In fact, when observing the trend of the survival function during certain follow-up intervals, the persistence of Group C was found to be superior to that of Group B (although lacking statistical significance in the analysis).

Therefore, we believe that our differences in persistence were not significantly influenced by the presence of the nocebo effect; rather, there were other related aspects that should be taken into account. On the other hand, the treatment-related adverse events are detailed in their corresponding section.

### 4.2. Subcutaneous Injection Devices and Health Education

In the context of subcutaneous injection devices, Group A consisted of patients who received various adalimumab biosimilar drugs with different application devices. This aspect has a negative impact on patients, as it causes heightened anxiety about the treatment. The specific drugs SB5, GP2017, and FKB327 are administered by physically pressing the device onto abdominal skin and maintaining pressure until the medication is injected. In contrast, ABP501 is administered by depressing the plunger of a prefilled syringe, similar to the administration method used for the original adalimumab (although the latter incorporates an autoinjector device). Generally, patients find it more uncomfortable to administer the drug using pressure devices compared with prefilled syringes. Applying sustained pressure on the abdomen during drug injection exacerbates discomfort and elicits more abdominal pain in these patients, who often experience abdominal pain due to their underlying condition. However, if this aspect were responsible for the observed differences in persistence, we would expect the results to favor Group B (original), but surprisingly, our obtained results were in the opposite direction.

Regarding the potential impact of discrepancies in health education, no such differences existed within our cohort, as all patients received instruction from the same nurse without differentiation based on their assigned groups. Health education plays a crucial role in addressing these concerns and ensuring patient comfort with novel devices, thereby minimizing the occurrence of the nocebo effect; improving adherence, tolerance, and efficacy; and ultimately leading to increased persistence. In conclusion, although Group A was the most heterogeneous in terms of the types of injectors used, our results suggest that this did not have a significant influence on the results, possibly due in part to the consistent health education provided to all patients by our reference nurse. This highlights the importance of health education in reinforcing adherence and treatment effectiveness.

### 4.3. Combined Therapy

While the effectiveness of the combination therapy involving adalimumab and immunosuppressants may not appear to be superior in terms of inducing and maintaining remission in Crohn’s disease (CD), a condition extensively evaluated in this type of treatment, it is imperative to acknowledge that this therapeutic regimen is linked with reduced immunogenicity [34]. Consequently, it has the potential to prolong the duration of persistence [35]. Within our study population, Group B had the highest number of patients receiving combined therapy, suggesting that our findings on persistence were expected to favor this group regardless. At this juncture, it is crucial to consider another crucial variable that significantly impacts persistence: the occurrence of adverse effects (refer to Section 4.5).

### 4.4. Heterogeneity between Groups

We do not acknowledge the presence of potential heterogeneity in the initial patient baseline conditions as a plausible factor contributing to the observed differences between the groups. This is supported by the results section (Section 3.2 and Section 3.3), which specifically states that such heterogeneity was eliminated through a preliminary statistical analysis of initial biochemical parameters, HBI, and pMayo.

### 4.5. The Adverse Effects Incidence with Biosimilars

Group A had a notable percentage of treatment-emergent adverse events (TEAEs), but these were primarily mild and had minimal impact on the overall condition of the patients, thus not necessitating the frequent discontinuation of medication. In contrast, the other groups (B and C) experienced higher incidences of severe adverse effects when considering the total number of adverse events. This factor contributed to the higher persistence observed in Group A compared with the other two groups.

The prevalence of at least one adverse event in our patient population (35.5%) aligned with the existing literature; however, it is essential to acknowledge the heterogeneity among studies when examining adverse effects, as they are often not categorized as severe or mild, which we find intriguing. Previous studies have typically only focused on adverse effects leading to treatment suspension or interruption. This is evident in a study by Derikx L. [16], where a large cohort of patients (n = 481) were analyzed over the course of a year, resulting in a TEAE percentage of 24.1% when only considering those requiring the withdrawal of the medication (which corresponded to severe TEAEs in our study). This proportion exceeded our recorded rate of serious adverse reactions, which stands at 16.34%.

In comparison to the findings of the VOLTAIRE-CD study published in the Lancet journal in 2021 [32], our study achieved a similar rate of severe treatment-emergent adverse events (TEAEs), with 15.64% (27/147) closely resembling our rate of 16.34%. However, it is important to note that the aforementioned study did not make a comparison between those exclusively using the biosimilar and those who switched from the original. Furthermore, it is worth mentioning that our study utilized multiple biosimilars, whereas the VOLTAIRE-CD study only utilized a single biosimilar (SB5).

There are additional real-life studies, focused on the use of the biosimilar adalimumab in inflammatory bowel disease (IBD), that have been conducted. One such study, which included 87 patients with Crohn’s disease (CD) undergoing treatment with ABP 501, reported an overall adverse effects proportion of 25.3% over a six-month follow-up period [11].

In another study involving a sample size of 186 patients who predominantly had CD, 50% of the sample was treated with SB5 [12], and no significant differences were found in the occurrence of adverse effects within a ten-week timeframe between the two cohorts (one treated with the original adalimumab and the other with the biosimilar, SB5). However, there were disparities in relation to injection pain, which was reported in 33% of the overall sample. Specifically, 52.3% of patients treated with the biosimilar experienced injection pain compared with only 15% of those treated with the original adalimumab.

In a study published in 2018 that focused on SB5 (the most commonly used biosimilar in our hospital), a total of 508 patients with rheumatoid arthritis were included [36]. The study identified adverse reactions in 35.8% of patients during a 13-month follow-up period, although only 0.7% of patients discontinued therapy due to these reactions.

Although our proportion of adverse reactions was similar to that of other studies, in our case, we discovered significant disparities in severe reactions. These severe reactions were more prevalent in the mixed group (16.3%), resulting in discontinuation and thus influencing the disparity in persistence between groups. This detail emphasizes the importance of conducting detailed analyses of various adverse reactions in relation to the treatment and the necessity to distinguish between serious and minor effects.

Moreover, the time variable is another crucial aspect when interpreting results. An adverse effect may initially be mild, but if it persists over time or occurs repeatedly in the long term, it could transform into a treatment-emergent adverse event (TEAE) that leads to discontinuation. This can significantly impact the patient’s compliance and confidence in the treatment.

In light of all these statements, we consider some of the strengths of our study to be the one-year follow-up period and the comprehensive and detailed description provided regarding the biosimilars’ safety, and we emphasize the importance of distinguishing between serious and mild adverse reactions. Additionally, we would like to underscore the fact that we utilized multiple biosimilars in the same patient, a factor not previously explored in previous studies. This detail is further discussed in the interchangeability section.

### 4.6. Aspects Related to Drug Interchangeability

When utilizing biosimilar medications in clinical practice, it is crucial to distinguish between the concepts of biosimilarity and interchangeability. It is erroneous to assume that all biosimilar drugs are interchangeable, as this can decrease tolerance and adherence, potentially resulting in treatment failure. Our data reveal that patients who switched to one or more biosimilars during follow-up (Group C) had a higher occurrence of serious adverse events within the one-year period compared with patients who did not switch brands. This had an impact on the durability of the treatment.

Certain centers have mistakenly assumed that biosimilarity equates to interchangeability, leading to the use of various adalimumab brands in the same patient for short durations. These are important factors surrounding biosimilars that necessitate our awareness of the associated clinical consequences. In the case of adalimumab, only one brand meets the criteria for biosimilarity and is also approved by the FDA as interchangeable [24].

To date, there is limited literature regarding patients who have undergone the administration of more than two ADA biosimilars during follow-up (referred to as multiswitched). Only one study was conducted on this modality of change with another anti-TNF (infliximab), as demonstrated in the PERFUSE study [37], where no safety differences were observed between groups.

In our study, as outlined in the results section, seven patients (6.73%) who received two or more biosimilars were included in our cohort. Interestingly, there was a higher percentage of these patients in Group C (12% of patients in the mixed group had undergone multiswitching). This characteristic may have been correlated with the elevated rate of treatment-emergent adverse events leading to treatment discontinuation in this group. Although this subgroup of patients is highly specific and opens a new path in expanding knowledge about the use of biosimilars, we consider it insufficient in size to draw extensive conclusions. Nevertheless, it underscores the necessity to analyze data from other cohorts in the same circumstance.

## 5. Conclusions

Our study demonstrates that adalimumab biosimilars show both safety and efficacy for IBD, in line with previous publications. However, to enhance our understanding, it is crucial to conduct further real-life studies with larger sample sizes and longer follow-up periods. These studies will allow for a more comprehensive evaluation of tolerance and safety, as these aspects are better understood over extended periods of time. Additionally, it is essential to consider the practice of using multiple biosimilars in the same patient and the concept of interchangeability. While these biosimilars have shown biosimilarity, it is important to recognize that not all of them are interchangeable. Understanding these differences is vital in clinical practice to prevent adverse effects and ensure treatment efficacy. Our real-world observations indicate that when transitioning from an original to a biosimilar or when using multiple biosimilars interchangeably, there may be an increased risk of adverse effects, ultimately impacting treatment outcomes.

## Figures and Tables

**Table 1:** Examined cohorts—demographic and essential clinical data.

Demographic and Clinical Characteristics of the Global Cohort			Baseline Biomarkers	
Age (years, mean)		41.9	CRP (mg/dL)	15.5
Sex (male: female)		1:1	Albumin (mg/dL)	4
Disease´s evolution (months, median)		94.73 95% Confidence Interval: 76.62 to 112.83	Faecal Calprotectin (FC)	1473
Smokers		Never: 52 (50%) Former smokers: 32 (30.8%) Unknown: 7 (6.7%)	Hemoglobin (g/dL)	12.2
Absence of alcohol consumption		75 (72.11%)	Major Disease Phenotypes (according to the Montreal Classification)	
Previous surgery		35 (33.7%)	Crohn´s Disease 76 (73.1%)	A2 (41.3%) L1 (31.7%) B1 (27.9%)
Perianal´s disease		18 (23.7%)	Ulcerative Colitis 28 (26.9%)	E3 (16.3 %) S2 (22.1 %)
Previous immunomodulators		78 (75%)		
Extraintestinal manifestations 26 (25%)		Rheumatologicals 16 (15.4%) Dermatologicals 10 (9.6%)		
**Demographic and Clinical Characteristics per Groups N (% of the Total Sample)**				
		Group A	Group B	Group C
Corticodependence 88 (84.6%)		41 (39.4%)	25 (24%)	22 (21.2%)
Previous anti-TNF 29 (27.9%)		12 (11.5%)	12 (11.5%)	5 (4.8%)
Baseline´s clinical indexes (mean)	CD (Harvey Bradshaw Index)	6	6.4	3.66
	UC (Mayo partial Index)	6.9	8.2	5.11

**Table 2:** ^1^ Mean. ^2^ Global mean: % reduction in baseline parameter. ^3^ Group mean: % reduction in baseline parameter. Overall and group clinical improvement were evaluated by calculating the mean reductions in the Harvey–Bradshaw Index (HBI) and the partial Mayo score (pMayo) at weeks 10 and 20. Laboratory values, including serum C-reactive protein (CRP) and fecal calprotectin (FC), were also analyzed. Baseline mean values for these parameters were determined for each group, and the percentage reductions from baseline were calculated for weeks 10 and 20.

				**Group Mean**		
				**A**	**B**	**C**
Response according clinical indices	Week 10 (44.23%) ^1^			41.3%	28.3%	30.4%
	Week 20 (33.65%) ^1^			51.4%	31.4%	17.2%
Response according to inflammation biomarkers	CRP (mg/dL)	Week 0	15.51 ^1^	14.27	16.82	16.93
		Week 10	11.74 ^1^; (−24.30) ^2^	5.12 (−64.12) ^3^	20.91 (+24) ^3^	9.19 (−45.71) ^3^
		Week 20	9.33 ^1^; (−39.84) ^2^	9.87 (−30.84) ^3^	9.56 (−43.16) ^3^	8.57 (−49.38) ^3^
	Calprotectin (mcg/g)	Week 0	1473 ^1^	2175.83	1696	2387.93
		Week 10	1507 ^1^ (+2.3%) ^2^	1119.32 (−48.55) ^3^	986 (−41.86) ^3^	2416.08 (+1.17) ^3^
		Week 20	1309 ^1^ (−11.13) ^2^	1391.88 (−36) ^3^	967.29 (−42.96) ^3^	1570 (−34.25) ^3^

**Table 3:** This overview provides information on both mild and serious adverse events, displaying the total percentages and distributions across three groups: Group A (biosimilar), Group B (originator adalimumab), and Group C (mixed group).

Mild Adverse Events 20 (19.23%)	Group A	Group B	Group C
Mild infections 4 (20%)	1 (25%)	1 (25%)	2 (50%)
Neurological 3 (15%)	3 (100%)	0 (0%)	0 (0%)
Dermatological 3 (15%)	2 (66.7%)	0 (0%)	1 (33.3%)
Arthalgia 4 (20%)	2 (50%)	0 (0%)	2 (50%)
Asthenia 6 (30%)	3 (50%)	2 (33.3%)	1 (16.7%)
None 84 (80.77%)	39 (46.4%)	26 (31%)	19 (22.6%)
Pearson´s Chi-sqared test: Chi^2^ = 8.822, d.f = 10, *p* = 0.55			
**Severe Adverse Events** **17 (16.3%)**	**Group A**	**Group B**	**Group C**
Neurological 1 (5.9%)	0 (0%)	0 (0%)	1 (100%)
Dermatological 8 (47%)	1 (12.5%)	1 (12.5%)	6 (75%)
Severe arthropathy 1 (5.9%)	0 (0%)	1 (100%)	0 (0%)
More than one TEAEs 7 (41.2%)	2 (28.6%)	3 (42.8%)	2 (28.6%)
None 87 (83.7%)	47 (54%)	24 (27.6%)	16 (18.4%)
Pearson´s Chi-sqared test: Chi^2^ = 20.148, d.f = 8, *p* = 0.009			

**Table 4:** Adalimumab survival probability by group. n Risk: number of patients at risk of presenting the event (discontinuation of the drug); n. event: number of discontinuations presented in that follow-up week; n. censor: censored data; surv: adalimumab survival probability; Std. err: standard error; upper and lower: confidence interval limit.

Group A							
Time	*n* Risk	*n* Event	*n* Censor	Surv	Std. err	upper	lower
4	50	0	1	1.000	0.000	1.000	1.000
8	49	1	0	0.980	0.021	1.000	0.941
10	48	0	2	0.980	0.021	1.000	0.941
16	46	1	2	0.958	0.030	1.000	0.903
20	43	0	4	0.958	0.030	1.000	0.903
21	39	1	0	0.934	0.040	1.000	0.864
22	38	0	1	0.934	0.040	1.000	0.864
24	37	1	0	0.908	0.048	0.999	0.826
26	36	0	2	0.908	0.048	0.999	0.826
28	34	1	2	0.882	0.057	0.986	0.789
32	31	0	6	0.882	0.057	0.986	0.789
36	25	0	5	0.882	0.057	0.986	0.789
40	20	0	8	0.882	0.057	0.986	0.789
44	12	0	1	0.882	0.057	0.986	0.789
46	11	1	0	0.802	0.111	0.996	0.645
48	10	0	10	0.802	0.111	0.996	0.645
**Group B**							
10	29	1	0	0.966	0.035	1.000	0.901
16	28	1	0	0.931	0.051	1.000	0.843
19	27	1	0	0.897	0.063	1.000	0.792
20	26	1	0	0.862	0.074	0.997	0.745
24	25	2	0	0.793	0.095	0.955	0.659
28	23	2	0	0.724	0.115	0.907	0.578
32	21	1	0	0.690	0.125	0.880	0.540
44	20	1	0	0.655	0.135	0.853	0.503
46	19	1	0	0.621	0.145	0.825	0.467
48	18	0	18	0.621	0.145	0.825	0.467
**Group C**							
8	25	1	0	0.960	0.041	1.000	0.886
17	24	1	0	0.920	0.059	1.000	0.820
20	23	1	1	0.880	0.074	1.000	0.761
24	21	0	1	0.880	0.074	1.000	0.761
28	20	1	0	0.836	0.090	0.997	0.701
32	19	1	0	0.792	0.105	0.973	0.645
36	18	2	0	0.704	0.134	0.915	0.541
40	16	1	1	0.660	0.149	0.883	0.493
46	14	1	0	0.613	0.166	0.849	0.442
48	13	0	13	0.613	0.166	0.849	0.442

## Data Availability

The data used for this study cannot be made available due to patient confidentiality, but the first publication of these data may be found at https://www.ecco-ibd.eu/publications/congress-abstracts/item/p529-adalimumab-persistence-and-its-biosimilar-in-inflammatory-bowel-disease-experience-in-a-tertiary-centre.html (accessed on 16 October 2023). It was accepted as a poster presentation in the 16th Congress of ECCO Virtual 2021, 2–3 and 8–10 July 2021.
